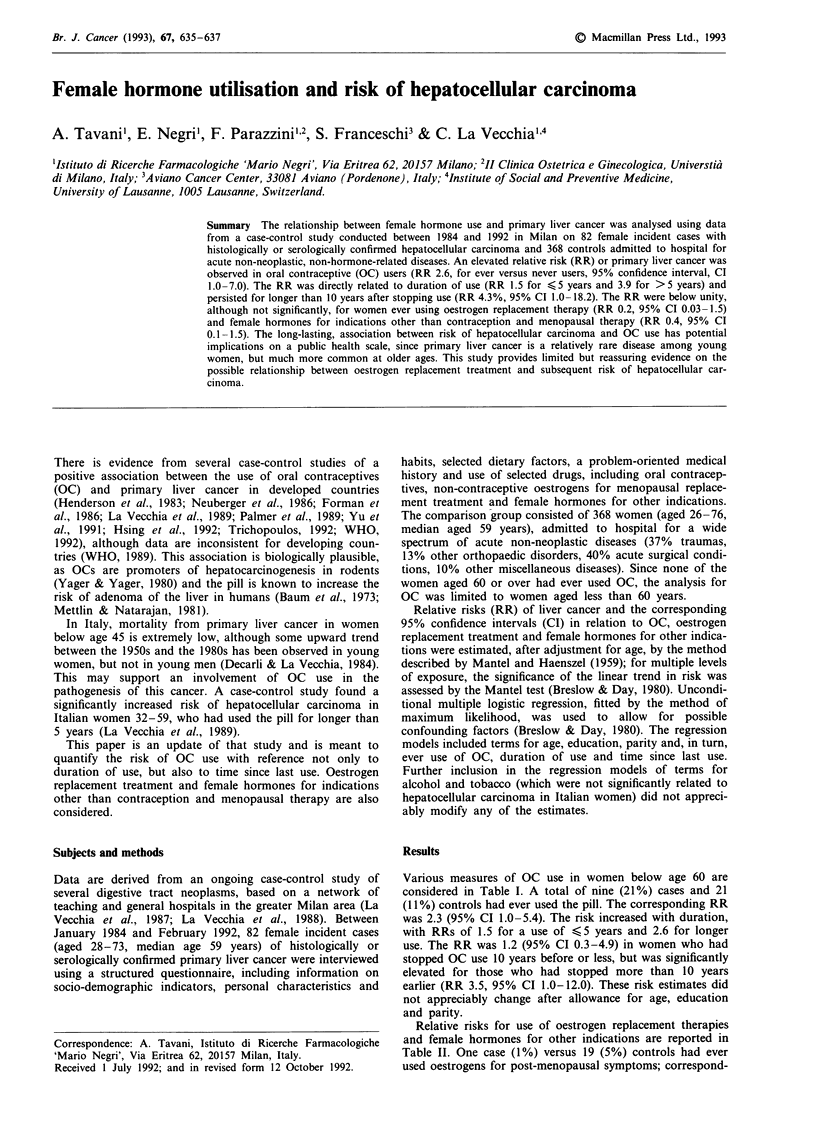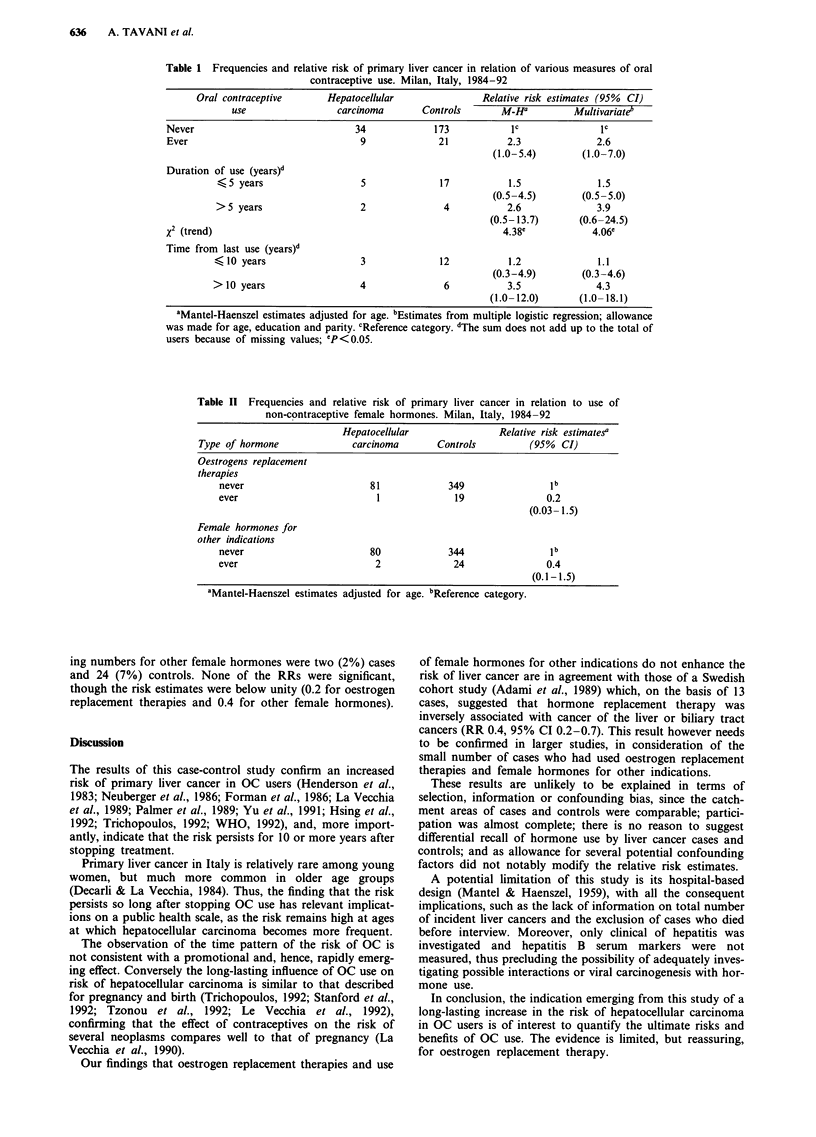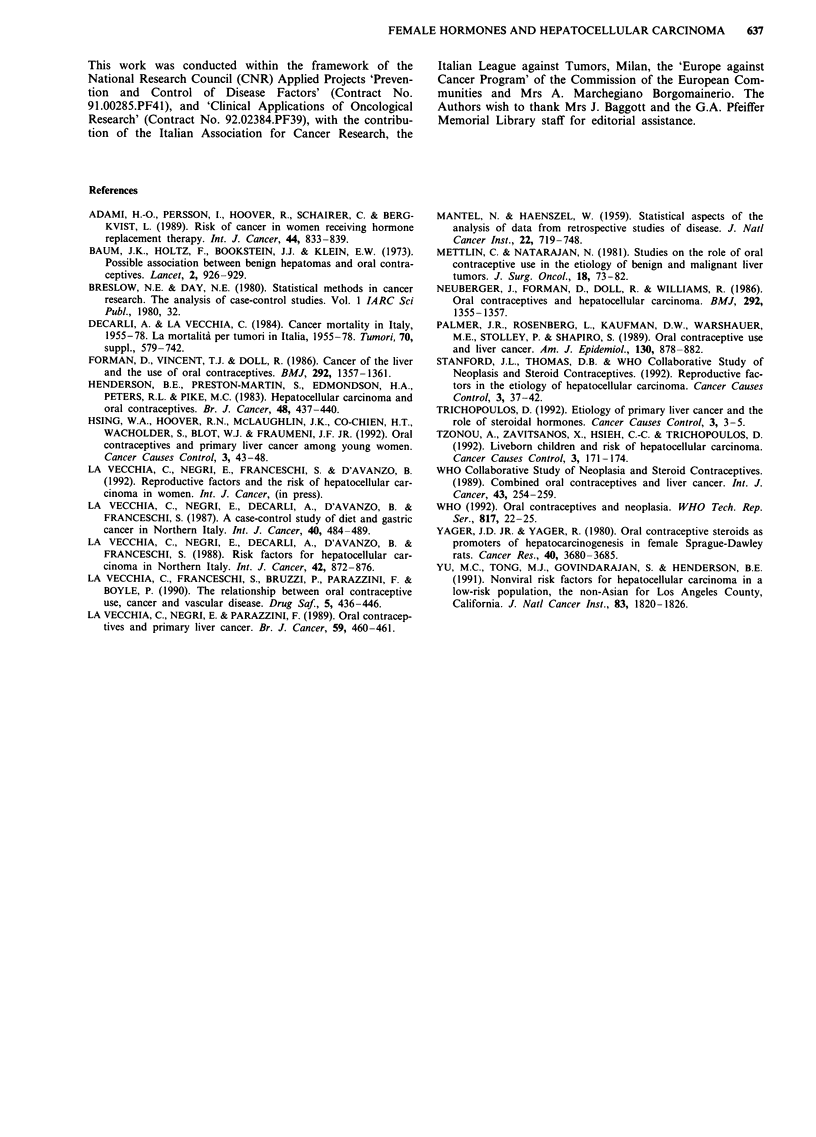# Female hormone utilisation and risk of hepatocellular carcinoma.

**DOI:** 10.1038/bjc.1993.116

**Published:** 1993-03

**Authors:** A. Tavani, E. Negri, F. Parazzini, S. Franceschi, C. La Vecchia

**Affiliations:** Istituto di Recherche Farmacologiche Mario Negri, Milano, Italy.

## Abstract

The relationship between female hormone use and primary liver cancer was analysed using data from a case-control study conducted between 1984 and 1992 in Milan on 82 female incident cases with histologically or serologically confirmed hepatocellular carcinoma and 368 controls admitted to hospital for acute non-neoplastic, non-hormone-related diseases. An elevated relative risk (RR) or primary liver cancer was observed in oral contraceptive (OC) users (RR 2.6, for ever versus never users, 95% confidence interval, CI 1.0-7.0). The RR was directly related to duration of use (RR 1.5 for < or = 5 years and 3.9 for > 5 years) and persisted for longer than 10 years after stopping use (RR 4.3%, 95% CI 1.0-18.2). The RR were below unity, although not significantly, for women ever using oestrogen replacement therapy (RR 0.2, 95% CI 0.03-1.5) and female hormones for indications other than contraception and menopausal therapy (RR 0.4, 95% CI 0.1-1.5). The long-lasting, association between risk of hepatocellular carcinoma and OC use has potential implications on a public health scale, since primary liver cancer is a relatively rare disease among young women, but much more common at older ages. This study provides limited but reassuring evidence on the possible relationship between oestrogen replacement treatment and subsequent risk of hepatocellular carcinoma.


Br. J. Cancer (1993), 67, 635-637 © Macmillan Press Ltd., 1993

### Female hormone utilisation and risk of hepatocellular carcinoma

A. Tavani[1], E. Negri[1], F. Parazzini[1,2], S. Franceschi[3] & C. La Vecchia[1,4]

[1]*Istituto di Ricerche Farmacologiche 'Mario Negri', Via Eritrea 62, 20157 Milano;* [2]*II Clinica Ostetrica e Ginecologica, Universtià di Milano, Italy;* [3]*Aviano Cancer Center, 33081 Aviano (Pordenone), Italy;* [4]*Institute of Social and Preventive Medicine, University of Lausanne, 1005 Lausanne, Switzerland.*

**Summary** The relationship between female hormone use and primary liver cancer was analysed using data from a case-control study conducted between 1984 and 1992 in Milan on 82 female incident cases with histologically or serologically confirmed hepatocellular carcinoma and 368 controls admitted to hospital for acute non-neoplastic, non-hormone-related diseases. An elevated relative risk (RR) or primary liver cancer was observed in oral contraceptive (OC) users (RR 2.6, for ever versus never users, 95% confidence interval, CI 1.0-7.0). The RR was directly related to duration of use (RR 1.5 for $\leqslant 5$ years and 3.9 for $>5$ years) and persisted for longer than 10 years after stopping use (RR 4.3%, 95% CI 1.0-18.2). The RR were below unity, although not significantly, for women ever using oestrogen replacement therapy (RR 0.2, 95% CI 0.03-1.5) and female hormones for indications other than contraception and menopausal therapy (RR 0.4, 95% CI 0.1-1.5). The long-lasting, association between risk of hepatocellular carcinoma and OC use has potential implications on a public health scale, since primary liver cancer is a relatively rare disease among young women, but much more common at older ages. This study provides limited but reassuring evidence on the possible relationship between oestrogen replacement treatment and subsequent risk of hepatocellular carcinoma.

There is evidence from several case-control studies of a positive association between the use of oral contraceptives (OC) and primary liver cancer in developed countries (Henderson *et al.*, 1983; Neuberger *et al.*, 1986; Forman *et al.*, 1986; La Vecchia *et al.*, 1989; Palmer *et al.*, 1989; Yu *et al.*, 1991; Hsing *et al.*, 1992; Trichopoulos, 1992; WHO, 1992), although data are inconsistent for developing countries (WHO, 1989). This association is biologically plausible, as OCs are promoters of hepatocarcinogenesis in rodents (Yager & Yager, 1980) and the pill is known to increase the risk of adenoma of the liver in humans (Baum *et al.*, 1973; Mettlin & Natarajan, 1981).

In Italy, mortality from primary liver cancer in women below age 45 is extremely low, although some upward trend between the 1950s and the 1980s has been observed in young women, but not in young men (Decarli & La Vecchia, 1984). This may support an involvement of OC use in the pathogenesis of this cancer. A case-control study found a significantly increased risk of hepatocellular carcinoma in Italian women 32-59, who had used the pill for longer than 5 years (La Vecchia *et al.*, 1989).

This paper is an update of that study and is meant to quantify the risk of OC use with reference not only to duration of use, but also to time since last use. Oestrogen replacement treatment and female hormones for indications other than contraception and menopausal therapy are also considered.

### Subjects and methods

Data are derived from an ongoing case-control study of several digestive tract neoplasms, based on a network of teaching and general hospitals in the greater Milan area (La Vecchia *et al.*, 1987; La Vecchia *et al.*, 1988). Between January 1984 and February 1992, 82 female incident cases (aged 28-73, median age 59 years) of histologically or serologically confirmed primary liver cancer were interviewed using a structured questionnaire, including information on socio-demographic indicators, personal characteristics and habits, selected dietary factors, a problem-oriented medical history and use of selected drugs, including oral contraceptives, non-contraceptive oestrogens for menopausal replacement treatment and female hormones for other indications. The comparison group consisted of 368 women (aged 26-76, median aged 59 years), admitted to hospital for a wide spectrum of acute non-neoplastic diseases (37% traumas, 13% other orthopaedic disorders, 40% acute surgical conditions, 10% other miscellaneous diseases). Since none of the women aged 60 or over had ever used OC, the analysis for OC was limited to women aged less than 60 years.

Relative risks (RR) of liver cancer and the corresponding 95% confidence intervals (CI) in relation to OC, oestrogen replacement treatment and female hormones for other indications were estimated, after adjustment for age, by the method described by Mantel and Haenszel (1959); for multiple levels of exposure, the significance of the linear trend in risk was assessed by the Mantel test (Breslow & Day, 1980). Unconditional multiple logistic regression, fitted by the method of maximum likelihood, was used to allow for possible confounding factors (Breslow & Day, 1980). The regression models included terms for age, education, parity and, in turn, ever use of OC, duration of use and time since last use. Further inclusion in the regression models of terms for alcohol and tobacco (which were not significantly related to hepatocellular carcinoma in Italian women) did not appreciably modify any of the estimates.

### Results

Various measures of OC use in women below age 60 are considered in Table I. A total of nine (21%) cases and 21 (11%) controls had ever used the pill. The corresponding RR was 2.3 (95% CI 1.0-5.4). The risk increased with duration, with RRs of 1.5 for a use of $\leqslant 5$ years and 2.6 for longer use. The RR was 1.2 (95% CI 0.3-4.9) in women who had stopped OC use 10 years before or less, but was significantly elevated for those who had stopped more than 10 years earlier (RR 3.5, 95% CI 1.0-12.0). These risk estimates did not appreciably change after allowance for age, education and parity.

Relative risks for use of oestrogen replacement therapies and female hormones for other indications are reported in Table II. One case (1%) versus 19 (5%) controls had ever used oestrogens for post-menopausal symptoms; correspond-

Correspondence: A. Tavani, Istituto di Ricerche Farmacologiche 'Mario Negri', Via Eritrea 62, 20157 Milan, Italy.
Received 1 July 1992; and in revised form 12 October 1992.

Table I Frequencies and relative risk of primary liver cancer in relation of various measures of oral contraceptive use. Milan, Italy, 1984–92

| *Oral contraceptive use* | *Hepatocellular carcinoma* | *Controls* | *Relative risk estimates (95% CI)* M-H[a] | *Multivariate*[b] |
|---|---|---|---|---|
| Never | 34 | 173 | 1[c] | 1[c] |
| Ever | 9 | 21 | 2.3 (1.0–5.4) | 2.6 (1.0–7.0) |
| Duration of use (years)[d] | | | | |
| ⩽5 years | 5 | 17 | 1.5 (0.5–4.5) | 1.5 (0.5–5.0) |
| >5 years | 2 | 4 | 2.6 (0.5–13.7) | 3.9 (0.6–24.5) |
| $\chi^2$ (trend) | | | 4.38[e] | 4.06[e] |
| Time from last use (years)[d] | | | | |
| ⩽10 years | 3 | 12 | 1.2 (0.3–4.9) | 1.1 (0.3–4.6) |
| >10 years | 4 | 6 | 3.5 (1.0–12.0) | 4.3 (1.0–18.1) |

[a]Mantel-Haenszel estimates adjusted for age. [b]Estimates from multiple logistic regression; allowance was made for age, education and parity. [c]Reference category. [d]The sum does not add up to the total of users because of missing values; [e]$P<0.05$.

Table II Frequencies and relative risk of primary liver cancer in relation to use of non-contraceptive female hormones. Milan, Italy, 1984–92

| *Type of hormone* | *Hepatocellular carcinoma* | *Controls* | *Relative risk estimates*[a] *(95% CI)* |
|---|---|---|---|
| *Oestrogens replacement therapies* | | | |
| never | 81 | 349 | 1[b] |
| ever | 1 | 19 | 0.2 (0.03–1.5) |
| *Female hormones for other indications* | | | |
| never | 80 | 344 | 1[b] |
| ever | 2 | 24 | 0.4 (0.1–1.5) |

[a]Mantel-Haenszel estimates adjusted for age. [b]Reference category.

ing numbers for other female hormones were two (2%) cases and 24 (7%) controls. None of the RRs were significant, though the risk estimates were below unity (0.2 for oestrogen replacement therapies and 0.4 for other female hormones).

### Discussion

The results of this case-control study confirm an increased risk of primary liver cancer in OC users (Henderson *et al.*, 1983; Neuberger *et al.*, 1986; Forman *et al.*, 1986; La Vecchia *et al.*, 1989; Palmer *et al.*, 1989; Yu *et al.*, 1991; Hsing *et al.*, 1992; Trichopoulos, 1992; WHO, 1992), and, more importantly, indicate that the risk persists for 10 or more years after stopping treatment.

Primary liver cancer in Italy is relatively rare among young women, but much more common in older age groups (Decarli & La Vecchia, 1984). Thus, the finding that the risk persists so long after stopping OC use has relevant implications on a public health scale, as the risk remains high at ages at which hepatocellular carcinoma becomes more frequent.

The observation of the time pattern of the risk of OC is not consistent with a promotional and, hence, rapidly emerging effect. Conversely the long-lasting influence of OC use on risk of hepatocellular carcinoma is similar to that described for pregnancy and birth (Trichopoulos, 1992; Stanford *et al.*, 1992; Tzonou *et al.*, 1992; Le Vecchia *et al.*, 1992), confirming that the effect of contraceptives on the risk of several neoplasms compares well to that of pregnancy (La Vecchia *et al.*, 1990).

Our findings that oestrogen replacement therapies and use of female hormones for other indications do not enhance the risk of liver cancer are in agreement with those of a Swedish cohort study (Adami *et al.*, 1989) which, on the basis of 13 cases, suggested that hormone replacement therapy was inversely associated with cancer of the liver or biliary tract cancers (RR 0.4, 95% CI 0.2–0.7). This result however needs to be confirmed in larger studies, in consideration of the small number of cases who had used oestrogen replacement therapies and female hormones for other indications.

These results are unlikely to be explained in terms of selection, information or confounding bias, since the catchment areas of cases and controls were comparable; participation was almost complete; there is no reason to suggest differential recall of hormone use by liver cancer cases and controls; and as allowance for several potential confounding factors did not notably modify the relative risk estimates.

A potential limitation of this study is its hospital-based design (Mantel & Haenszel, 1959), with all the consequent implications, such as the lack of information on total number of incident liver cancers and the exclusion of cases who died before interview. Moreover, only clinical of hepatitis was investigated and hepatitis B serum markers were not measured, thus precluding the possibility of adequately investigating possible interactions or viral carcinogenesis with hormone use.

In conclusion, the indication emerging from this study of a long-lasting increase in the risk of hepatocellular carcinoma in OC users is of interest to quantify the ultimate risks and benefits of OC use. The evidence is limited, but reassuring, for oestrogen replacement therapy.

This work was conducted within the framework of the National Research Council (CNR) Applied Projects 'Prevention and Control of Disease Factors' (Contract No. 91.00285.PF41), and 'Clinical Applications of Oncological Research' (Contract No. 92.02384.PF39), with the contribution of the Italian Association for Cancer Research, the Italian League against Tumors, Milan, the 'Europe against Cancer Program' of the Commission of the European Communities and Mrs A. Marchegiano Borgomainerio. The Authors wish to thank Mrs J. Baggott and the G.A. Pfeiffer Memorial Library staff for editorial assistance.

### References

ADAMI, H.-O., PERSSON, I., HOOVER, R., SCHAIRER, C. & BERGKVIST, L. (1989). Risk of cancer in women receiving hormone replacement therapy. *Int. J. Cancer*, **44**, 833-839.

BAUM, J.K., HOLTZ, F., BOOKSTEIN, J.J. & KLEIN, E.W. (1973). Possible association between benign hepatomas and oral contraceptives. *Lancet*, **2**, 926-929.

BRESLOW, N.E. & DAY, N.E. (1980). Statistical methods in cancer research. The analysis of case-control studies. Vol. 1 *IARC Sci Publ.*, 1980, 32.

DECARLI, A. & LA VECCHIA, C. (1984). Cancer mortality in Italy, 1955-78. La mortalità per tumori in Italia, 1955-78. *Tumori*, **70**, suppl., 579-742.

FORMAN, D., VINCENT, T.J. & DOLL, R. (1986). Cancer of the liver and the use of oral contraceptives. *BMJ*, **292**, 1357-1361.

HENDERSON, B.E., PRESTON-MARTIN, S., EDMONDSON, H.A., PETERS, R.L. & PIKE, M.C. (1983). Hepatocellular carcinoma and oral contraceptives. *Br. J. Cancer*, **48**, 437-440.

HSING, W.A., HOOVER, R.N., MCLAUGHLIN, J.K., CO-CHIEN, H.T., WACHOLDER, S., BLOT, W.J. & FRAUMENI, J.F. JR. (1992). Oral contraceptives and primary liver cancer among young women. *Cancer Causes Control*, **3**, 43-48.

LA VECCHIA, C., NEGRI, E., FRANCESCHI, S. & D'AVANZO, B. (1992). Reproductive factors and the risk of hepatocellular carcinoma in women. *Int. J. Cancer*, (in press).

LA VECCHIA, C., NEGRI, E., DECARLI, A., D'AVANZO, B. & FRANCESCHI, S. (1987). A case-control study of diet and gastric cancer in Northern Italy. *Int. J. Cancer*, **40**, 484-489.

LA VECCHIA, C., NEGRI, E., DECARLI, A., D'AVANZO, B. & FRANCESCHI, S. (1988). Risk factors for hepatocellular carcinoma in Northern Italy. *Int. J. Cancer*, **42**, 872-876.

LA VECCHIA, C., FRANCESCHI, S., BRUZZI, P., PARAZZINI, F. & BOYLE, P. (1990). The relationship between oral contraceptive use, cancer and vascular disease. *Drug Saf.*, **5**, 436-446.

LA VECCHIA, C., NEGRI, E. & PARAZZINI, F. (1989). Oral contraceptives and primary liver cancer. *Br. J. Cancer*, **59**, 460-461.

MANTEL, N. & HAENSZEL, W. (1959). Statistical aspects of the analysis of data from retrospective studies of disease. *J. Natl Cancer Inst.*, **22**, 719-748.

METTLIN, C. & NATARAJAN, N. (1981). Studies on the role of oral contraceptive use in the etiology of benign and malignant liver tumors. *J. Surg. Oncol.*, **18**, 73-82.

NEUBERGER, J., FORMAN, D., DOLL, R. & WILLIAMS, R. (1986). Oral contraceptives and hepatocellular carcinoma. *BMJ*, **292**, 1355-1357.

PALMER, J.R., ROSENBERG, L., KAUFMAN, D.W., WARSHAUER, M.E., STOLLEY, P. & SHAPIRO, S. (1989). Oral contraceptive use and liver cancer. *Am. J. Epidemiol.*, **130**, 878-882.

STANFORD, J.L., THOMAS, D.B. & WHO Collaborative Study of Neoplasis and Steroid Contraceptives. (1992). Reproductive factors in the etiology of hepatocellular carcinoma. *Cancer Causes Control*, **3**, 37-42.

TRICHOPOULOS, D. (1992). Etiology of primary liver cancer and the role of steroidal hormones. *Cancer Causes Control*, **3**, 3-5.

TZONOU, A., ZAVITSANOS, X., HSIEH, C.-C. & TRICHOPOULOS, D. (1992). Liveborn children and risk of hepatocellular carcinoma. *Cancer Causes Control*, **3**, 171-174.

WHO Collaborative Study of Neoplasia and Steroid Contraceptives. (1989). Combined oral contraceptives and liver cancer. *Int. J. Cancer*, **43**, 254-259.

WHO (1992). Oral contraceptives and neoplasia. *WHO Tech. Rep. Ser.*, **817**, 22-25.

YAGER, J.D. JR. & YAGER, R. (1980). Oral contraceptive steroids as promoters of hepatocarcinogenesis in female Sprague-Dawley rats. *Cancer Res.*, **40**, 3680-3685.

YU, M.C., TONG, M.J., GOVINDARAJAN, S. & HENDERSON, B.E. (1991). Nonviral risk factors for hepatocellular carcinoma in a low-risk population, the non-Asian for Los Angeles County, California. *J. Natl Cancer Inst.*, **83**, 1820-1826.